# Mechanistic insights on 1-butene polymerization catalyzed by homogeneous single-site catalysts: a DFT computational study

**DOI:** 10.3389/fchem.2024.1377740

**Published:** 2024-03-13

**Authors:** Olga D’Anania, Claudio De Rosa, Giovanni Talarico

**Affiliations:** ^1^ Scuola Superiore Meridionale, Napoli, Italy; ^2^ Dipartimento di Scienze Chimiche, Università degli Studi di Napoli Federico II, Napoli, Italy; ^3^ Scuola Normale Superiore, Pisa, Italy

**Keywords:** olefin polymerization, DFT calculations, stereoselective polymerization, 1-butene polymerization, transition metal catalysis

## Abstract

Isotactic poly (1-butene) (iPB) is an interesting semi-crystalline thermoplastic material characterized by notable physical and mechanical attributes encompassing superior creep and stress resistance, elevated toughness, stiffness, and thermal endurance. These distinctive features position iPB as a viable candidate for specific applications; however, its widespread utilization is hindered by certain inherent limitations. Indeed, iPB manifests an intricate polymorphic behavior, and the gradual and spontaneous transition of the kinetically favored form II to the thermodynamically favored form I during aging introduces alterations to the material’s properties. Despite its potential, the attainment of iPB with an exceedingly high molecular mass remains elusive, particularly when employing homogeneous catalysts renowned for their efficacy in propene polymerization. In this study we analyze the mechanistic aspects governing 1-butene polymerization by using DFT calculations modelling the regioselectivity of 1-butene insertions and the termination reactions occurring after primary (1,2) and secondary (2,1) insertions. Finally, the isomerization pathways leading to the formation of 4,1 units in iPB samples synthesized by homogenous catalysts is also discussed. All these aspects, furnish a mechanistic picture of the main drawbacks of an “old” but still interesting material.

## Introduction

Polyolefins can be regarded as the most important class of polymeric materials produced by industries, accounting for more than half of the total weight of the globally produced polymers (Olabisi, 2016; Sauter et al., 2017; Talarico et al., 2019). Indeed, they are well-suited for a broad spectrum of applications that cover practically every aspect of our everyday lives (Hutley and Ouederni, 2016). Among these, polyethylene (PE) and polypropylene (PP) occupy the largest market share (Makaryan and Sedov, 2020). However, there exist other noteworthy α-olefin-based materials with peculiar characteristics. Notably, isotactic poly (1-butene) (iPB), a semi-crystalline thermoplastic material synthesized commercially using Ziegler-Natta catalysts (Natta et al., 1955), exhibits several remarkable physical and mechanical properties surpassing those of PE and PP. iPB’s commendable attributes, including superior creep and stress resistance, high toughness, stiffness, and tear strength (Luciani et al., 1988), position it as the material of choice for diverse applications ranging from hot water pipes to pressurized tanks and food packaging. However, its widespread industrial use is constrained by certain drawbacks, primarily associated with its complex polymorphic behavior.

Upon crystallization from the melt, iPB adopts the kinetically favored form II (Turner-Jones, 1963; Petraccone et al., 1976), gradually and spontaneously transforming into the thermodynamically favored form I at room temperature (Natta et al., 1960; De Rosa et al., 2009a; De Rosa et al., 2009b). These distinct crystalline forms possess disparate intrinsic properties (density, melting point, etc.), impacting material characteristics, particularly mechanical performance (Nakamura et al., 1999).

Effective control of the form II-form I transformation rate holds importance from both academic and industrial perspectives (Qiao et al., 2016; Tashiro et al., 2016). Substantial efforts have been dedicated to addressing this challenge, such as the introduction of defects in the iPB main chain through comonomeric units (Gianotti and Capizzi, 1969; De Rosa et al., 2019; De Rosa et al., 2020a) or stereoerrors (Schaffhauser, 1967; De Rosa et al., 2014). Another challenge lies in the realm of homogeneous catalysis, where certain complexes known for their efficacy in propene polymerization exhibit disparate behavior when applied to 1-butene polymerization, particularly concerning molecular weight capability.

**CHART 1:**
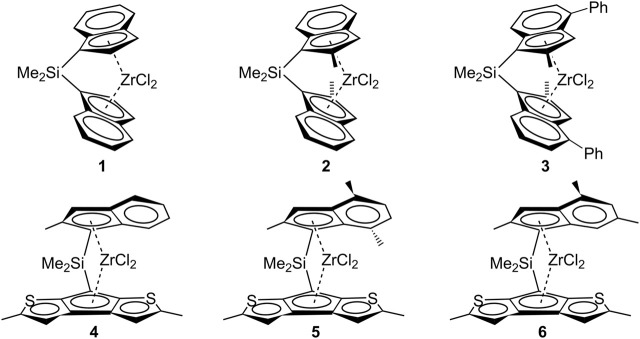
*C*_2_- and *C*_1_-symmetric metallocene systems employed for 1-butene polymerization.

Illustratively, the *C*_2_-symmetric Me_2_Si(Ind)_2_ZrCl_2_ metallocene system **1** (Chart 1) yields iPB with a low molecular weight (*M*_v_ = 16,000, at a polymerization temperature of 50°C) in the case of 1-butene polymerization (Resconi et al., 2006). Similarly, substituted *C*_2_-symmetric metallocenes, like Me_2_Si(2-Me-Ind)_2_ZrCl_2_ (system **2**) and Me_2_Si(2-Me-4-Ph-Ind)_2_ZrCl_2_ (system **3**), known for improved propene polymerization performance, do not exhibit equivalent efficacy in 1-butene polymerization (*M*_v_ = 381,100 at a polymerization temperature of 50°C for system **2** and *M*_v_ = 111,000 at a polymerization temperature of 70°C for system **3**).

Notwithstanding, Resconi and coworkers reported instances of fully regioselective 1-butene polymerization by using heterocycle *C*_1_-metallocene systems (Chart 1, systems **4**–**6**) that yield high molecular weight iPB (Resconi et al., 2006).

Numerous investigations have underscored the notable influence of regioerrors (or 2,1 insertions) on the propagation rate in propene polymerization (Busico et al., 1998; Correa et al., 2007). Indeed, due to the difficulties encountered by the monomer insertion into a secondary growing chain, a low reactive “dormant site” is formed (Jüngling et al., 1995; Landis et al., 2004; Busico et al., 2005). The study by Resconi et al. (2006) suggests that regioirregular insertions can exert a profound impact on 1-butene polymerization, influencing the balance between propagation and termination reactions.

It is noteworthy that 1-butene polymerization is a multifaceted process characterized by side mechanisms that give rise to a distinctive polymer microstructure, differing in several aspects from that of polypropylene. First, the analysis of ^13^C NMR spectra indicates that the majority of metallocene catalysts allow the occasional incorporation of 2,1 units in the polypropylene main chain (Resconi et al., 2000). Notably, in the case of iPB samples, the presence of 2,1 units has exclusively been identified at chain ends, with none detected in the main chain (Busico et al., 1995).

Second, the microstructure of polypropylene samples obtained by metallocenes may be characterized by the presence of 3,1 units, resulting from an isomerization of the 2,1 units (Resconi, 1999). In contrast, the presence of 3,1 units has not been observed for iPB. Instead, various studies have consistently reported the detection of 4,1-units (Busico et al., 1995; Borriello et al., 1997), attributed to the isomerization of the 2,1 unit.

**SCHEME 1:**
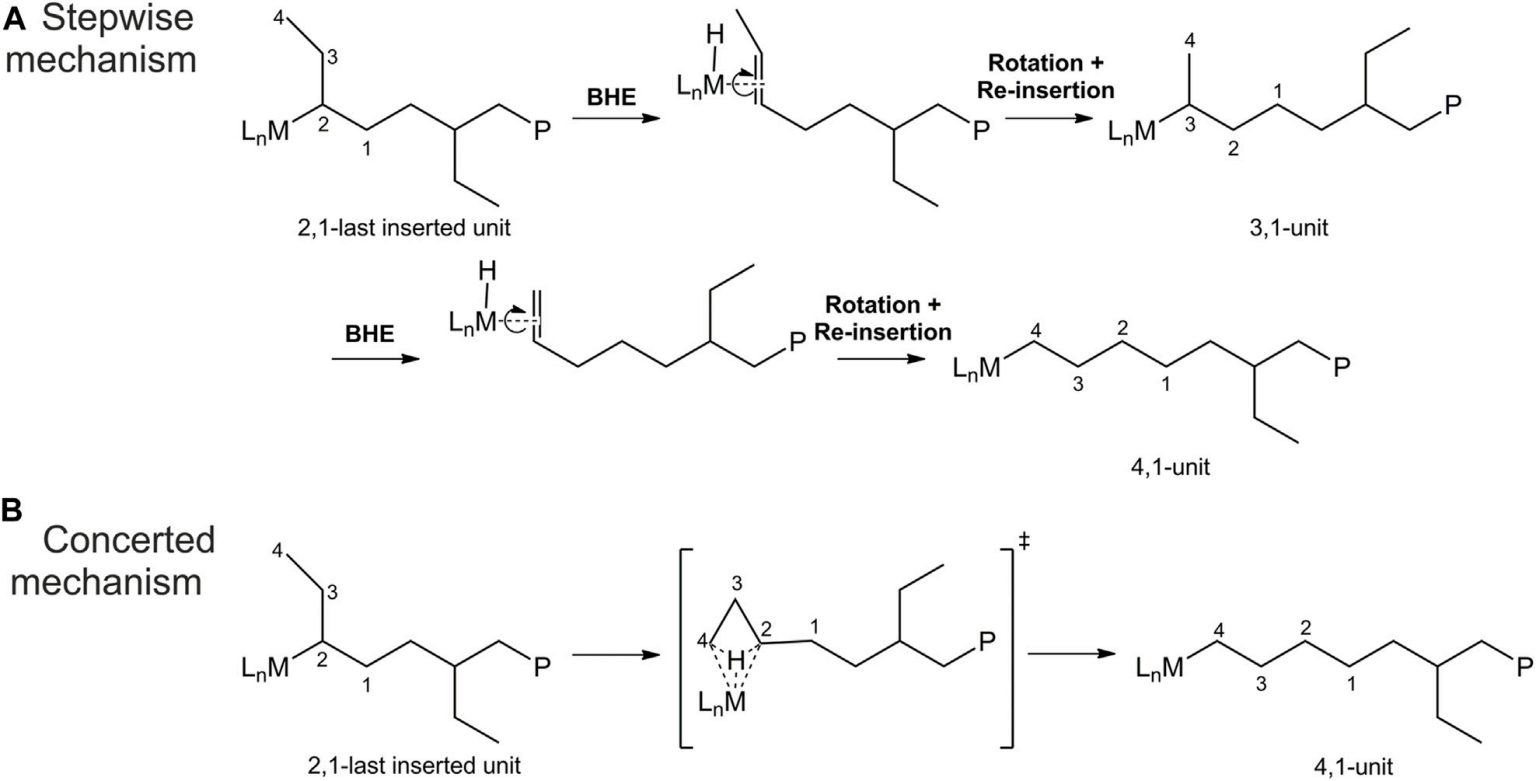
Mechanisms for the stepwise **(A)** and concerted **(B)** isomerization of 2,1-last inserted butene units leading to 4,1 units.

Finally, the isomerization path of 2,1 butene units remains uncertain, with two proposed mechanisms: a stepwise mechanism and a concerted mechanism (Busico et al., 1995). The stepwise mechanism (Scheme 1A) involves a sequence of β-H transfer to the metal, the formation of a double bond at the chain end, rotation of the olefin, and re-insertion into the metal-hydrogen bond. This process leads to the formation of a 3,1-unit intermediate, and the steps are reiterated, ultimately yielding the 4,1-unit product. In addition to this proposed mechanism, as 3,1 units have never been detected, a concerted mechanism (Scheme 1B) has also been suggested. The concerted mechanism involves a single transition state (TS) and directly produces the 4,1 unit from the 2,1-last inserted butene unit.

While propene polymerization has been extensively investigated in various aspects, including stereoselectivity (Corradini et al., 1979; Corradini et al., 2004; Busico et al., 2006), regioselectivity (Busico et al., 1998; Talarico et al., 2003; Busico et al., 2004; Correa et al., 2007), competition between propagation and termination reactions (Talarico et al., 2002; Talarico and Budzelaar, 2006; Caporaso et al., 2010), and isomerization mechanisms (Busico and Cipullo, 1995; Pilme et al., 2007), a comprehensive theoretical exploration focused on the mechanistic aspects of 1-butene polymerization is notably absent. To address this gap, we embarked on an in-depth analysis of 1-butene polymerization through the application of density functional theory (DFT) calculations.

The limited outcomes observed for iPB samples produced by *C*_2_-symmetric metallocene catalysts, particularly in terms of molecular weight, have been systematically investigated. This elucidation involves an examination of the stereoselectivity and regioselectivity in 1-butene polymerization, the termination reactions following primary (1,2) and secondary (2,1) insertions, and an exploration of both stepwise and concerted isomerization pathways leading to 4,1 units in the iPB main chain.

For the sake of simplicity, DFT calculations have been carried out on the prototype *C*_2_-symmetric *ansa*-metallocene system **1**, although the obtained results are easily extended to other types of *C*_2_-symmetric metallocene catalysts.

## Computational details

The structures of the transition states (TSs) and intermediates have been optimized by using the Gaussian16 set of programs (Frisch et al., 2016) and the B3LYP hybrid Generalized Gradient Approximation (GGA) functional (Becke, 1988; Lee et al., 1988) combined with two layers of basis set has been selected. The standard polarized split-valence basis set of Ahlrichs and co-workers (SVP) (Schäfer et al., 1992) has been used for H, C, and Si, meanwhile the LANL2DZ basis and Effective Core Potential (ECP) (Hay and Wadt, 1985) have been employed for the metal centre. The nature of the stationary points has been determined by performing a vibrational analysis and by checking the presence of zero or only one imaginary frequency for intermediates or TSs, respectively. This analysis has been also used for the computation of zero-point energies and thermal (enthalpy and entropy) corrections (298.15 K, 1 bar). Refined electronic energies have been obtained by performing single point calculations with B3LYP functional and a larger TZVP basis set for H, C, and Si (Weigend et al., 2003) and the SDD basis augmented with a f function with exponent 0.5 and pseudopotential (Wadt and Hay, 1985) for Zr. The dispersion corrections (EmpiricalDispersion = D3) (Grimme et al., 2010) and solvation contribution (toluene) through the Polarizable Continuum Model (PCM) (Cossi et al., 1996) have been added. These refined electronic energies have been added to the thermal corrections computed at the SVP/LANL2DZ to obtain the Δ*G* hereafter reported. This computational approach has been already tested in the olefin polymerization catalysis and found to be viable (Falivene et al., 2015; Falivene et al., 2018). In the following we define: the stereoselectivity Δ*E* (Δ*G* ^#^_stereo_) as the electronic (Gibbs energy) difference between the favored TSs for 1,2 insertions with different enantiofaces; the regioselectivity Δ*E* (Δ*G* ^#^_regio_) as the electronic (Gibbs energy) difference between the favored TSs for 1,2 and 2,1 monomer insertions; the competition between propagation and termination reactions Δ*E* (Δ*G* ^#^_T-P_) as the electronic (Gibbs energy) between the favored α-olefin insertion (Δ*G*_P_) and the lower β-H transfer to the monomer (Δ*G*_T_). Positive values of Δ*G* ^#^_T-P_ indicate that propagation is favored over termination. For 1-butene polymerization, calculations have been performed by employing a -CH_2_CH(CH_2_CH_3_)CH_2_CH_2_CH_3_ group in order to reproduce the primary growing chain, instead the secondary chain, formed by a 2,1-last inserted butene unit, is simulated by a -CH(CH_2_CH_3_)CH_2_CH_2_CH_2_CH_3_ group. For propene polymerization, instead, calculations with ^*i*^Bu group simulating the primary growing chain and a ^*s*^Bu group simulating a secondary chain with the 2,1-last inserted propene unit have been used.

## Results and discussion

The results of DFT calculations on the mechanistic aspects of 1-butene polymerization promoted by catalyst **1** are summarized in Table 1, including data obtained from propene polymerization for comparative analysis.

**TABLE 1:** Calculated Δ*E* (Δ*G*)^*#*^ in kcal/mol for stereoselectivity, regioselectivity and competition between termination and propagation reactions for propene and 1-butene polymerization promoted by system **1**.

Monomer	Chain	Δ*E* (Δ*G*)^#^_stereo_ ^a^	Δ*E* (Δ*G*)^#^_regio_ ^a^	Δ*E* (ΔG)^#^_T-P_ ^b^
Propene	Primary	4.2 (4.4) kcal/mol	3.3 (3.1) kcal/mol	6.7 (4.4) kcal/mol
	Secondary	1.4 (0.6) kcal/mol	2.1 (1.3) kcal/mol	2.6 (0.6) kcal/mol
1-Butene	Primary	4.7 (3.8) kcal/mol	3.1 (2.7) kcal/mol	7.1 (4.6) kcal/mol
	Secondary	0.0 (0.1) kcal/mol	1.3 (0.5) kcal/mol	−0.4 (−2.7) kcal/mol

^a^ The Δ*E* (Δ*G*)^#^_stereo_ and Δ*E* (Δ*G* ^#^)_regio_ values are calculated with respect to the favored monomer enantioface TSs.

^b^ The Δ*E* (ΔG)^#^_T-P_ values are obtained by comparing the most stable TS for BHT (as reported in the main text) with the favored monomer enantioface insertion TS into the growing polymer chain.

The calculated stereoselectivities on a primary growing chain reveal a remarkable similarity between propene and 1-butene as indicated by the Δ*E* (Δ*G*)^#^_stereo_ values in Table 1 (Mercandelli et al., 2007). This congruence aligns with the established concept that stereocontrol originates from the chiral orientation of the growing chain, as elucidated by (Corradini et al., 2004). The TS structures, depicted in Supplementary Figure S1, show the stabilization of the chiral conformation of the polymer chain and DFT calculation highlights the pivotal role of α-agostic interaction in enhancing the stability of the chiral arrangement for both propene and 1-butene monomers (Talarico and Budzelaar, 2016). Furthermore, the utilization of a secondary growing chain results in a substantial loss of stereoselectivity, consistent with the proposed model. The close resemblance between the TS structures for propene and 1-butene in this context (Supplementary Figure S2) is indicative of Gibbs energies approaching zero (0.6 kcal/mol for propene and 0.1 kcal/mol for 1-butene).

DFT calculations of the regioselectivity observed in the polymerization of propene and 1-butene, is still emphasizing the similarity between the two monomers (Ratanasak et al., 2021). The Δ*E* (Δ*G*)^#^_regio_ values in Table 1 underscore the consistent regioselectivity in both cases. It is well-established that 2,1 insertions are unlikely to occur in α-olefin polymerization catalyzed by metallocene catalysts, attributed to electronic and steric factors, (Correa et al., 2007). The energetic profiles of the TS structures for propene and 1-butene on a primary chain, as depicted in Supplementary Figure S3, exhibit striking similarities. A nuanced analysis of regioselectivity with a secondary chain reveals a smaller variation for propene (1.3 kcal/mol) compared to almost complete loss for 1-butene (0.5 kcal/mol), as illustrated in Supplementary Figure S4. Moving from a primary to a secondary chain results in a substantial decrease in regioselectivity, measuring 1.8 kcal/mol for propene and 2.2 kcal/mol for 1-butene. The impact of the secondary chain is more pronounced in 1-butene polymerization, where the more encumbered ethyl group interacts with the 2,1-last inserted unit (Supplementary Figure S4).

Overall, the calculated values for Δ*E* (Δ*G*)^#^_stereo_ and Δ*E* (Δ*G*)^#^_regio_ of Table 1 with primary and/or secondary growing chain still did not account for the peculiarity of the 1-butene polymerization kinetics as well as the microstructures reported experimentally for the two α-olefins highlighted in the Introduction. So, we decided to investigate the termination reactions and their energetic comparison with the propagation steps.

**FIGURE 1:**
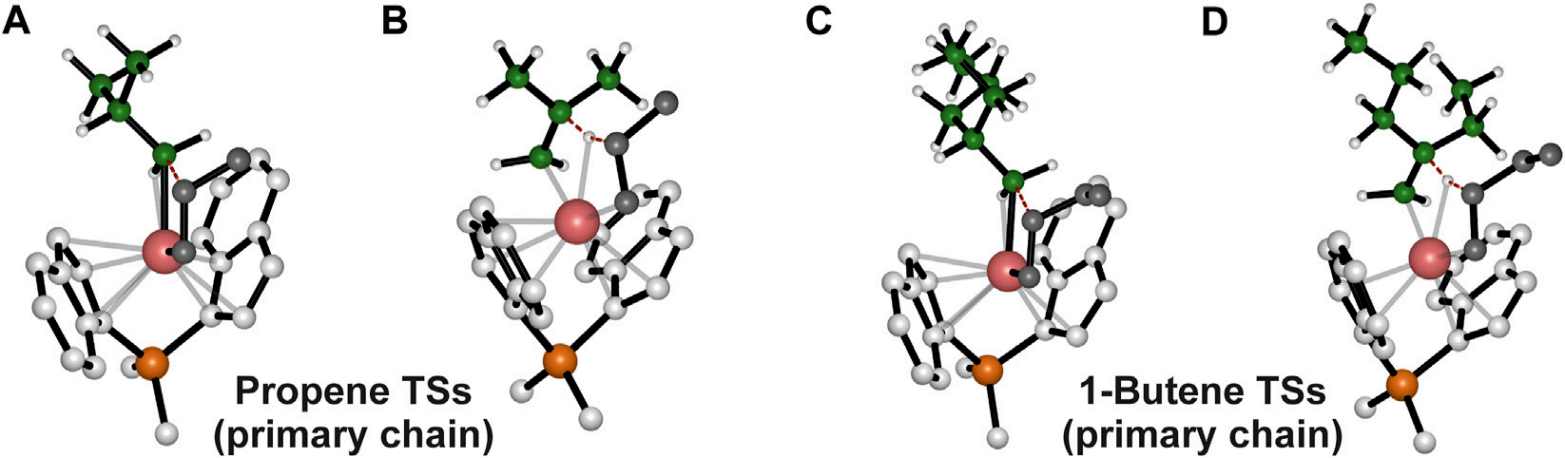
DFT optimized structures of the preferred propene **(A)** and 1-butene **(C)** insertion into a primary growing polymer chain TSs and the β-H transfer from the primary chain to propene **(B)** and 1-butene **(D)** monomers TSs. The hydrogen atoms belonging to the monomer and the ligand are omitted for simplicity. The growing polymer chain and the monomer are represented in green and grey, respectively.

Our initial analysis involves a comparison of the preferred TSs for propagation and termination reactions, considering the active species with a primary chain derived from a 1,2-last inserted unit. The chain release can occur via two types of β-hydrogen transfers: β-H transfer to the monomer (BHT) and β-hydrogen elimination (BHE), where a β-H is transferred from the growing polymer chain to the metal (Cavallo and Guerra, 1996). For system **1** the BHT plays a fundamental role in lowering the molecular weight of the obtained polymer. As a matter of fact, modifications of the ligand framework by adding a Me substituent at the position 2 of the indenyl group destabilize the spatially demanding 6-centers TS of the BHT with respect to the 4-centers TS of the propagation and samples characterized by higher molecular weight are obtained (Stehling et al., 1994). Therefore, the free energies of the preferred TSs with propene and 1-butene insertion into a primary growing polymer chain (Figures 1A, C) and their favoured TSs for the termination via BHT from a primary chain (Figures 1B, D), are compared (Laine et al., 2010). The calculated Δ*E* (Δ*G*)^#^_T-P_ reported in Table 1 show that the TS for BHT is higher in free energy than for propagation and still the propene (ΔG^#^_T-P_ = 4.4. kcal/mol) and 1-butene (ΔG^#^_T-P_ = 4.6 kcal/mol) results predict homopolymers with similar molecular mass if termination reaction occurs by a primary growing chain.

Taking into account that, as already mentioned in the introduction section, more regioselective metallocene complexes seem to lead to iPB characterized by higher molecular weight (Resconi et al., 2006), we hyphotesized a direct correlation between the occurrence of regioerrors and molecular mass so we extended our study to the analysis of species bearing a secondary chain, formed by a 2,1-last inserted unit.

**SCHEME 2:**
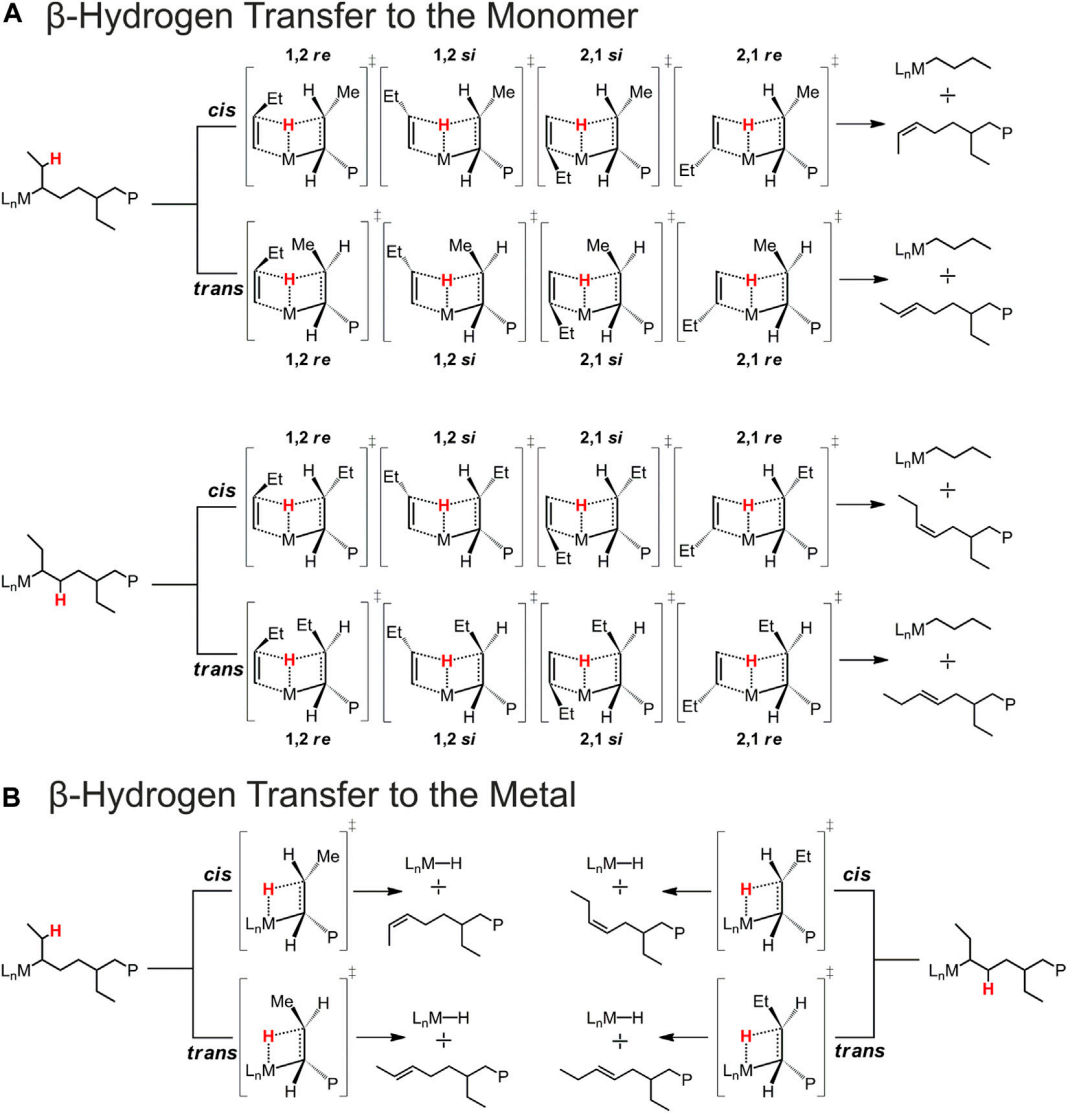
Schematic representation of the possible BHT and BHE TSs occurring after a 2,1 1-butene insertion leading to different chain end groups. **(A)** β-Hydrogen transfer to the monomer. **(B)** β-Hydrogen transfer to the metal.

It is important to highlight that termination reactions occurring in the presence of a 2,1-last inserted unit lead to a much more complicated *scenario* with respect to the case of a primary growing chain. In fact, for a secondary chain, there are two β-hydrogens prone to be transferred to the monomer (BHT) or to the metal (BHE) belonging to two different methylene groups, the one of the main chain and the other of the side ethyl group. Moreover, both BHT and BHE give an internal double bond, which can be *cis* or *trans* in configuration (Scheme 2).

The most relevant BHT and BHE TS structures for 1-butene polymerization are reported in Supplementary Figures S5, S6, and their electronic (Gibbs) energies values are reported in Supplementary Tables S1, S2.

**FIGURE 2:**
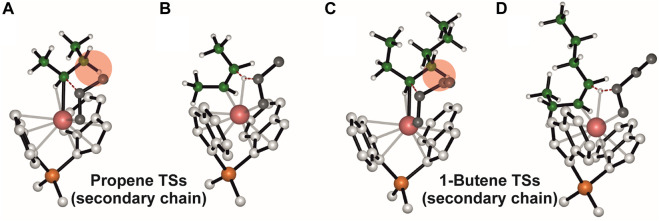
DFT optimized structures of the “right” propene **(A)** and 1-butene **(C)** enantioface insertion into a secondary growing polymer chain TSs and the β-H transfer from the secondary chain to (1,2) propene **(B)** and 1-butene **(D)** TSs. The hydrogen atoms belonging to the monomer and the ligand are omitted for simplicity. The growing polymer chain and the monomer are represented in green and grey, respectively.

Interestingly, we found that the Δ*E* (Δ*G*)^#^_T-P_ for propene polymerization by a secondary chain is still positive (2.6 (0.6) kcal/mol, Table 1) favoring the propagation (Figure 2A) over the termination (Figure 2B) (Caporaso et al., 2010). The calculated Δ*E* (Δ*G*)^#^_T-P_ for 1-butene changes dramatically and the insertion (Figure 2C) is highly disfavored with respect to the termination (Figure 2D) with a (Δ*G*)^#^_T-P_ = −2.7 kcal//mol. The preferred BHT corresponds to the β-H transfer from the methylene of the main chain to the 1,2 1-butene (Figure 2D) and it is the most stable among the analyzed BHT TSs since both the alkyl groups linked to the forming double C-C bond are oriented in the less hindered area of the ligand framework. Furthermore, this TS leads to the formation of a *cis*-3,4-pentenyl chain end group, which has been experimentally observed through ^13^C NMR spectra analysis (Resconi et al., 2006).

Therefore, it can be stated that when a 1-butene regioirregular unit is inserted, the termination step is favored over propagation. Catalysts that are not fully regioselective give low molecular weight iPB, due to the synergistic effect of a destabilized 1-butene insertion (monomer-chain interaction) and a stabilized termination TSs.

In fact, it is noteworthy that β-H transfer from a primary growing chain TS, which is higher in energy than the respective propagation TS, is characterized by a disfavoring interaction between the monomer and the side ethyl group (Figure 1D), instead no destabilizing interactions are present in the TS in which the β-H is transferred from a secondary chain. Hence, differently from what happens with 1-butene, 2,1 propene units can be incorporated in the main chain and high molecular weight polymers are obtained also by employing catalysts not fully regioselective.

**FIGURE 3:**
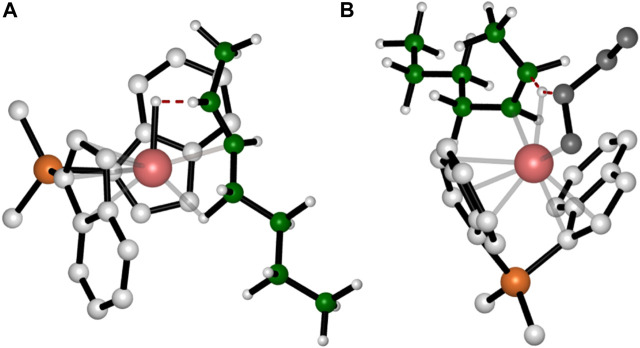
DFT optimized structures of the favorite TSs for BHE **(A)** and BHT **(B)** occurring after the epimerization of the secondary chain. For simplicity the hydrogen atoms belonging to the monomer and the ligand are omitted. The growing polymer chain and the monomer are represented in green and grey, respectively.

Concerning the TSs for the BHE reactions, we found that the most stable TS is the one reported in Figure 3A, which corresponds to a β-H transfer from the ethyl side group to the metal. It should yield a *trans*-2,3-pentenyl chain end group, which, however, has never been detected experimentally. In fact, they can be considered as the starting point for the stepwise isomerization mechanism leading to 4,1 units in the iPB main chain.

**FIGURE 4:**
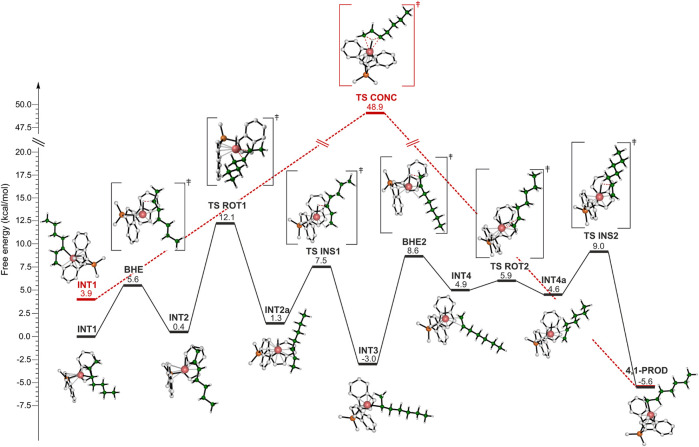
DFT calculated energy profile for the stepwise (black) and concerted (red) isomerization mechanisms leading to 4,1 units.

As already mentioned in the introduction section, the isomerization mechanism is still uncertain and both a stepwise and a concerted mechanism have been proposed (Busico et al., 1995). DFT calculations have been employed to evaluate both the mechanisms. Figure 4 reports the energy profiles where the pathway for the stepwise isomerization mechanism is represented in black, and the concerted one is shown in red. Let us first discuss the stepwise mechanism. The reference point (INT1) is the catalyst which bears a secondary growing polymer chain stabilized by a β-agostic interaction between the metal and the H atom of the methylene belonging to the ethyl side group. INT1 evolves into the TS determining the first BHE of the reaction pathway: the H involved in the β-agostic is transferred to the Zr atom, an extra β-agostic interaction contributes to the stabilization of the TS. The *trans* configuration of the growing chain yields an intermediate (INT2) showing a *trans* 2,3 C=C bond (INT2). At this point, a 180° rotation of the olefin around the metal center occurs. The TS for this rotation (TS ROT1) was found to be the highest energy TS of the whole isomerization path, thus being the rate determining step (rds). It is destabilized by the unfavorable orientation of the olefin, indeed both the alkyl groups (Me and *n*-Butyl groups) linked to the C=C bond point towards the indenyl ligands. TS ROT1 leads to INT2a, which is about 1 kcal/mol higher in energy with respect to the previous intermediate. The reaction proceeds with the reinsertion of the rotated olefin into the Zr-H bond (TS INS1) and the formation of INT3 intermediate. INT3 corresponds to a 3,1 unit and shows a β-agostic interaction between a hydrogen from the methyl group and the metal atom. It evolves in the TS BHE2 by the H transfer to the metal center and into the formation of a terminal double bond (INT4). Also in this case, an additional β-agostic interaction enables the stabilization of the TS. Again, the rotation of the formed double bond occurs. After the rotation, re-insertion of the unsaturated terminal unit into the Zr-H bond takes place and the 4,1 unit is formed.

It is important to highlight that both thermodynamics and kinetics account for the lack of 3,1-units in the main chain of iPB. The 4,1-unit product is more stable than the 3,1-unit and the whole activation energy of the path from 3,1 to 4,1-unit is comparable with the rds energy involved in the formation of the 3,1-unit (Figure 4).

**FIGURE 5:**
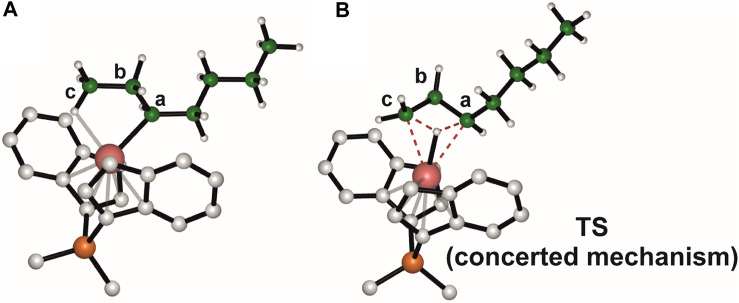
DFT optimized geometries for the γ-agostic secondary chain intermediate **(A)** and the TS governing the concerted isomerization mechanism **(B)**.

The structures of the species involved in the concerted mechanism are reported for clarity also in Figure 5. The intermediate (Figure 5A) displays a γ-agostic interaction that involves a hydrogen of the methyl group and precedes the TS (Figure 5B), which gives directly the 4,1 product from a 2,1-last inserted unit. The hydrogen belonging to the carbon atom **c** is transferred to the carbon **a**, meanwhile the hydrogen atom interacts with the metal center and the bond between the zirconium and carbon **c** is formed. Although the concerted mechanism seems easier than the stepwise one, DFT calculations indicate that the TS involved in the former is 36.8 kcal/mol higher in free energy than the TS governing the rate limiting step of the latter (TS ROT1).

The 3,1-unit is not observed in the iPB main chain likely due to the kinetics and thermodynamics of the stepwise isomerization path, which result in its transformation in the 4,1-unit, rather than the occurrence of a concerted isomerization mechanism.

Therefore, once a 2,1-unit is inserted, either termination occurs, or a 4,1-unit is formed, and a primary alkyl group linked to the metal center is obtained. In the latter case the propagation can continue and the molecular weight of the achieved PB is slightly higher than what expected from the ΔΔ*G* ^#^_T-P_ value.

**FIGURE 6:**
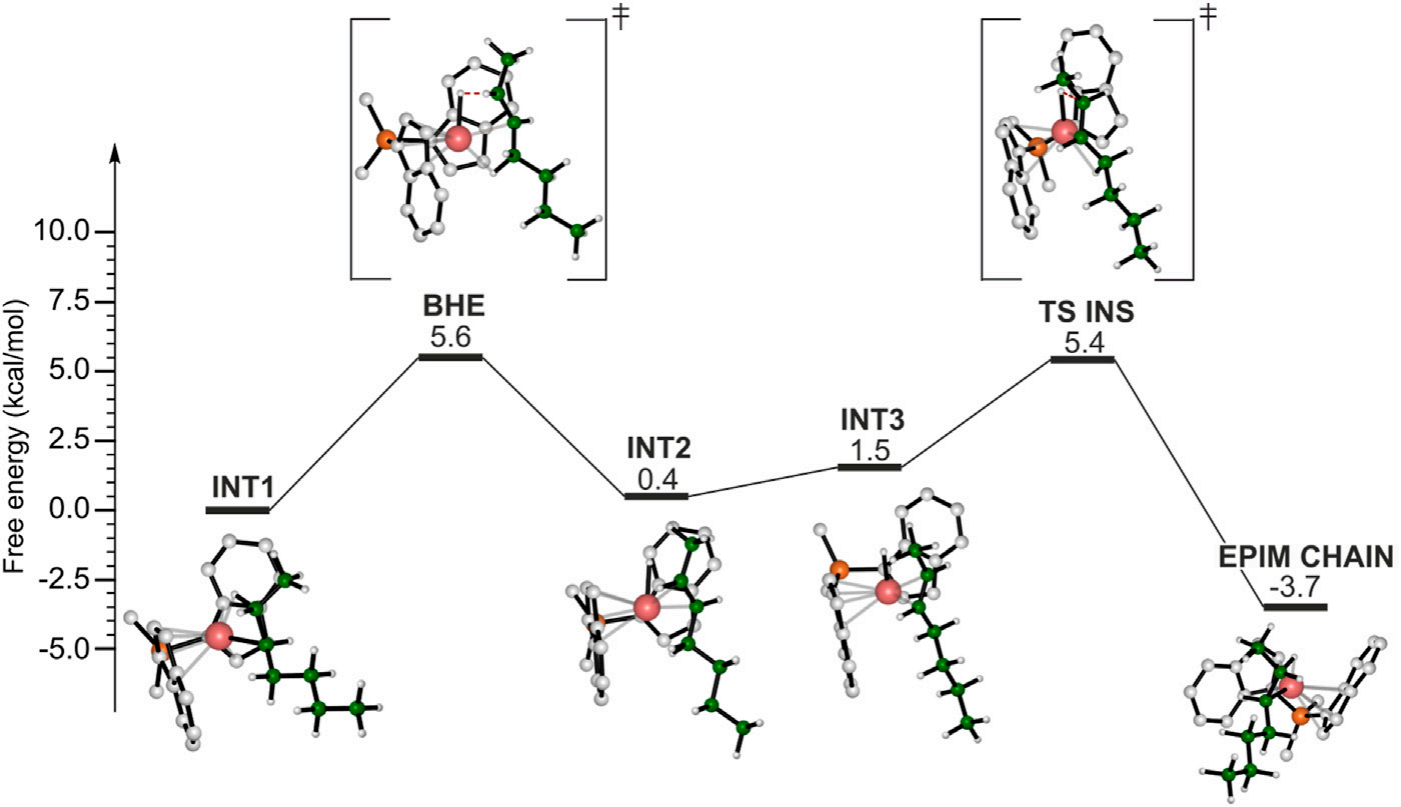
DFT calculated energy profile for the epimerization of the secondary chain.

Finally, we hypothesized that epimerization of the secondary chain may provide an alternative route to isomerization. The calculated DFT energy profile is shown in Figure 6. The reference point is the same as the isomerization and the BHE consists in the H transfer from the methylene of the ethyl side group of the chain leading to the INT2 intermediate, displaying a double bond in *trans* configuration. At this point the epimerization reaction pathway differs from the isomerization one: in the latter the formed double bond rotates around the metal center, instead, in this case the olefin reinserts with the opposite enantioface (TS INS) and a secondary chain characterized by an inversed configuration of the chiral carbon atom is formed (EPIM CHAIN). The epimerization is likely to occur due to its thermodynamic driving force (Figure 6).

Once the epimerization has taken place, termination is still preferred over propagation. In fact, we found that the favored BHT occurring after the epimerization of the secondary chain involves the transfer of the β-H belonging to the methylene of the ethyl group to the 1,2 *re* butene enantioface, exhibits the incoming C=C bond in a *cis* configuration (Figure 3B) and is 3.0 kcal/mol lower in energy with respect to the favored butene insertion TS (see Supplementary Figure S7).

The occurrence of the epimerization of the secondary chain followed by the BHT governed by the aforementioned TS is corroborated by the ^13^C NMR analysis, which indicates also the presence of *cis*-2,3-pentenyl chain end groups (Resconi et al., 2006). The structures of the relevant BHT TSs from the epimerized chain and their relative energies are reported in Supplementary Figure S8; Supplementary Table S3, respectively.

## Conclusion

In this work a comprehensive mechanistic study of 1-butene polymerization promoted by a *C*_2_-symmetric *ansa*-metallocene prototype complex has been carried out by using DFT calculations.

Our findings offer valuable insights into the impact of regioerrors on 1-butene kinetics (Kim et al., 2021), revealing a preference for chain termination from a secondary growing chain over propagation. This observation elucidates the lower molecular weight observed in isotactic polybutene (iPB) samples produced by the *ansa*-metallocene systems.

Upon the insertion of a 2,1 butene unit, our study identifies an alternative route to termination reactions through the isomerization of regioirregular units. Both stepwise and concerted mechanisms, previously proposed in the literature, have been modeled. DFT results consistently support the stepwise mechanism as the most feasible, highlighting the formation of 3,1-units that convert into more thermodynamically stable 4,1-units.

These insights are not confined to the specific *C*_2_-symmetric metallocene system studied herein but are extendable to other analogous systems. The presented findings lay the groundwork for future endeavors focused on enhancing the molecular weight of iPB samples, with the hopes to enlarge the polymer molecular architectures as already reported for iPP (De Rosa et al., 2017; De Rosa et al., 2018; De Rosa et al., 2020b) overcoming the main drawbacks of an “old” but still interesting material (Han et al., 2022).

## Data Availability

The raw data supporting the conclusion of this article will be made available by the authors, without undue reservation.
