# Supplementary material for: Mechanistic insights on 1-butene polymerization catalyzed by homogeneous single-site catalysts: a DFT computational study
Source: Front Chem. 2024 Mar 13;12:1377740. doi: 10.3389/fchem.2024.1377740 (PMC10965545; doi:10.3389/fchem.2024.1377740)
Supplement: Supplementary file 1 [file DataSheet1.PDF]

Supplementary Material

Mechanistic insights on 1-butene polymerization catalyzed by homogeneous single-site catalysts: a DFT computational study

Olga D'Anania,[1,2,3] Claudio De Rosa[2] and Giovanni Talarico[1,2] *

[1] Scuola Superiore Meridionale, Largo San Marcellino 10, 80138 Napoli, Italy
[2] Dipartimento di Scienze Chimiche, Università degli Studi di Napoli Federico II, Via Cintia, 80126 Napoli, Italy
[3] Scuola Normale Superiore, Piazza dei Cavalieri 7, 56126 Pisa, Italy

CONTENT

Figure S1 2

Figure S2 3

Figure S3 4

Figure S4 5

Figure S5 6

Figure S6 7

Table S1 8

Table S2 8

Figure S7 9

Figure S8 10

Table S3 11

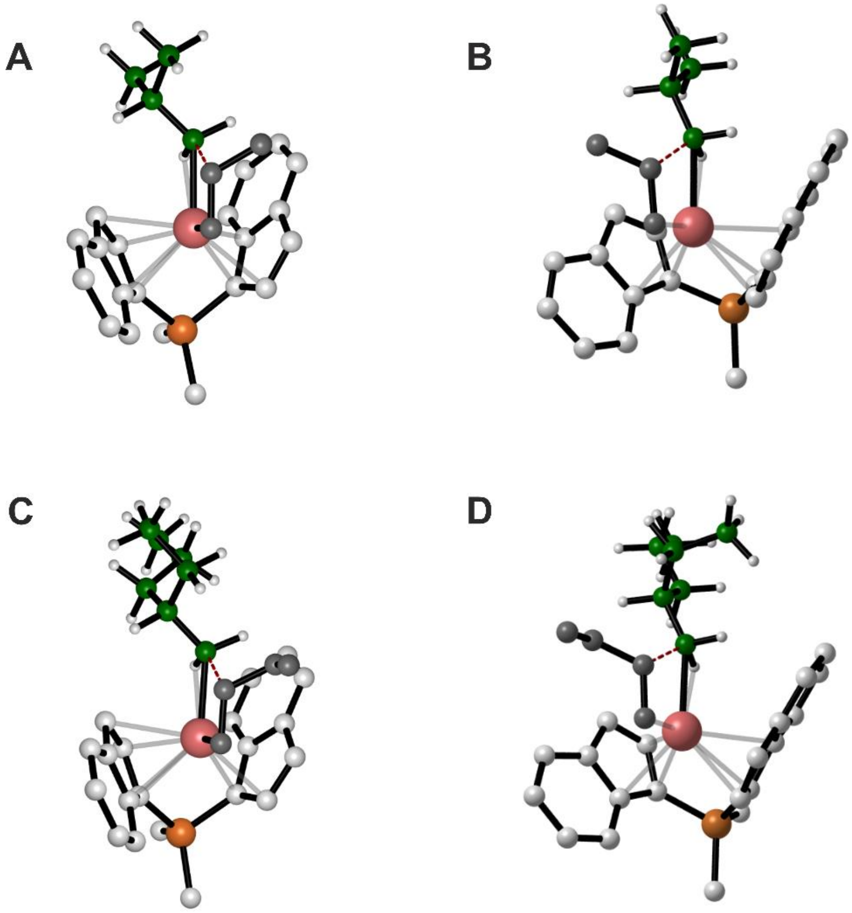

Figure S1. Optimized structures for the 1,2 propene insertion with different enantiofaces (A, B) and the analogous 1-butene insertion (C, D) to calculate the $\Delta E(\Delta G)^{\#}_{stereo}$ with a primary growing chain. The hydrogen atoms of the ligand and monomer are omitted for clarity. The monomer and the growing chain are reported in grey and green, respectively.

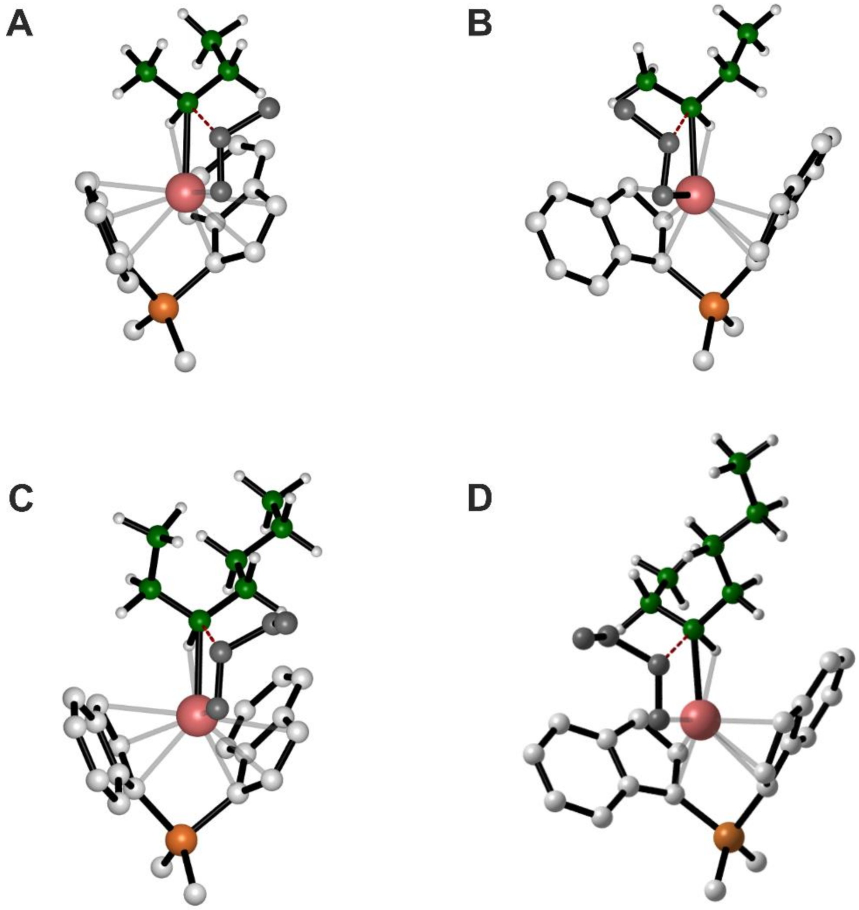

Figure S2. Optimized structures for the 1,2 propene insertion with different enantiofaces (A, B) and the analogous 1-butene insertion (C, D) to calculate the $\Delta E(\Delta G)^{\#}_{\mathrm{stereo}}$ with a secondary growing chain. The hydrogen atoms of the ligand and monomer are omitted for clarity. The monomer and the growing chain are reported in grey and green, respectively.

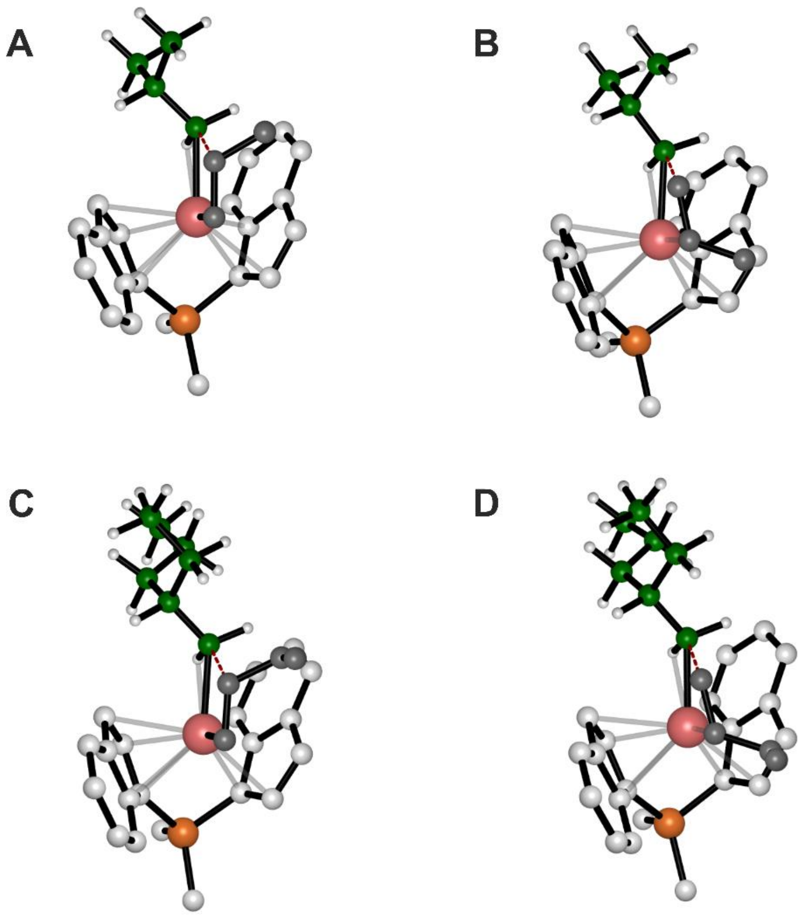

Figure S3. Optimized structures for 1,2 and 2,1 propene insertion (A, B) and the analogous 1-butene insertion (C, D) to calculate the $\Delta E(\Delta G)^{\#}_{regio}$ with a primary growing chain. The hydrogen atoms of the ligand and monomer are omitted for clarity. The monomer and the growing chain are reported in grey and green, respectively.

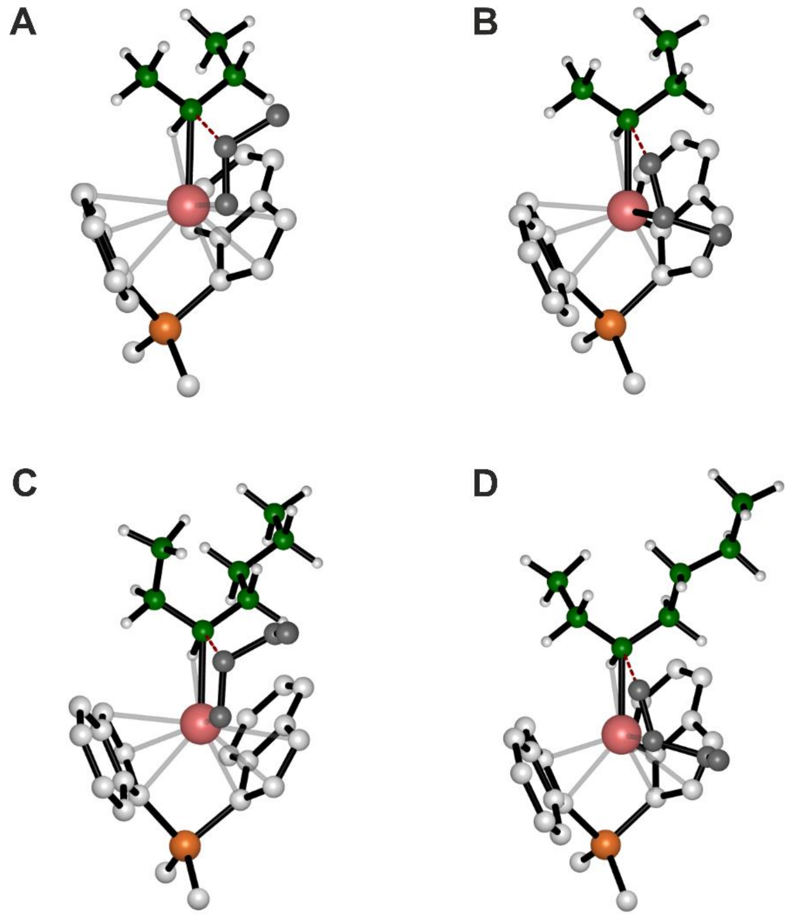

Figure S4. Optimized structures for 1,2 and 2,1 propene insertion (A, B) and the analogous 1-butene insertion (C, D) to calculate the $\Delta E(\Delta G)^{\#}_{regio}$ with a secondary growing chain. The hydrogen atoms of the ligand and monomer are omitted for clarity. The monomer and the growing chain are reported in grey and green, respectively.

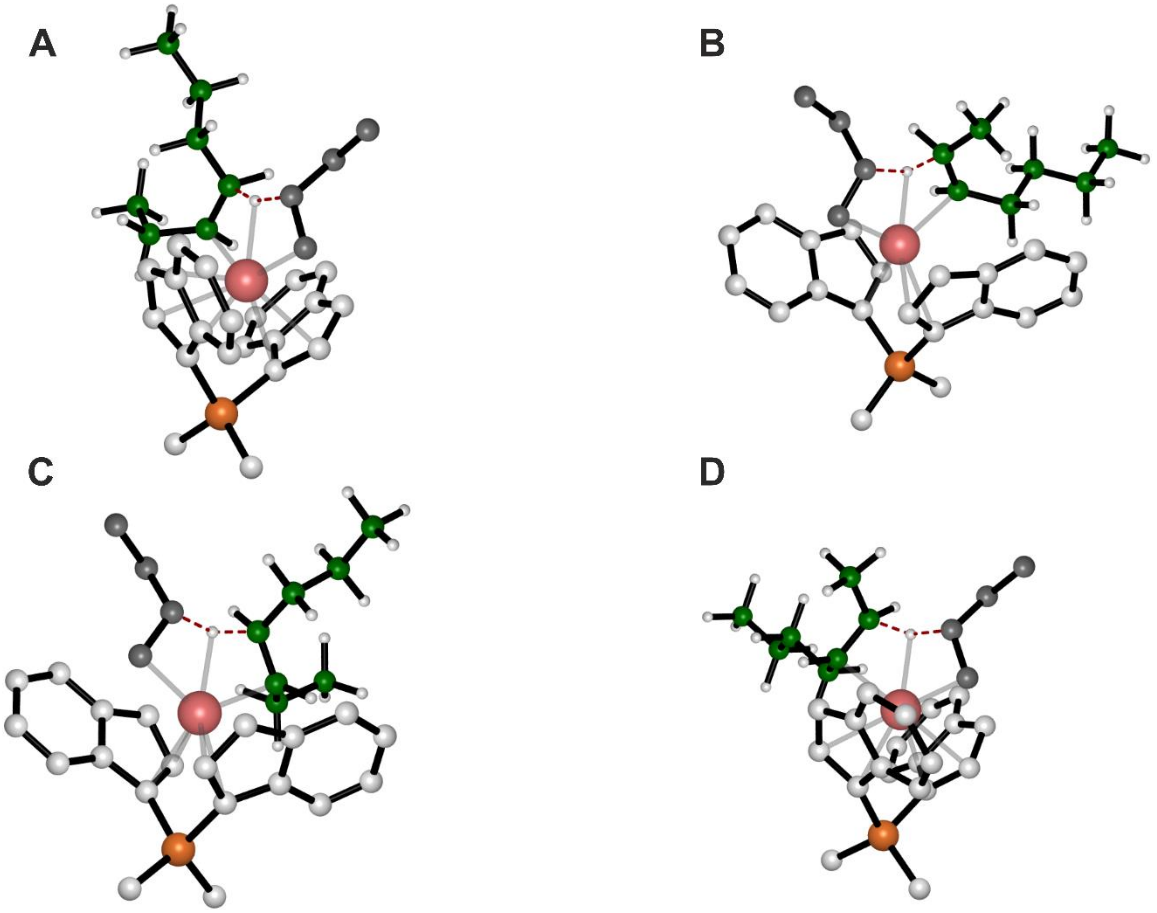

Figure S5. Optimized structures for the relevant BHT TSs occurring after a 2,1 butene insertion. (A) β-H transfer from the methylene of the chain to 1,2 *re* butene enantioface, the forming C=C is in a *cis* configuration. (B) β-H transfer from the methylene of the ethyl side group to 1,2 *si* butene enantioface, the forming C=C shows *cis* configuration. (C) β-H transfer from the methylene of the main chain to 1,2 *si* butene, the forming C=C exhibits a *trans* configuration. (D) β-H transfer from the methylene of the ethyl side group to 1,2 *re* butene enantioface, the forming C=C displays a *trans* configuration.

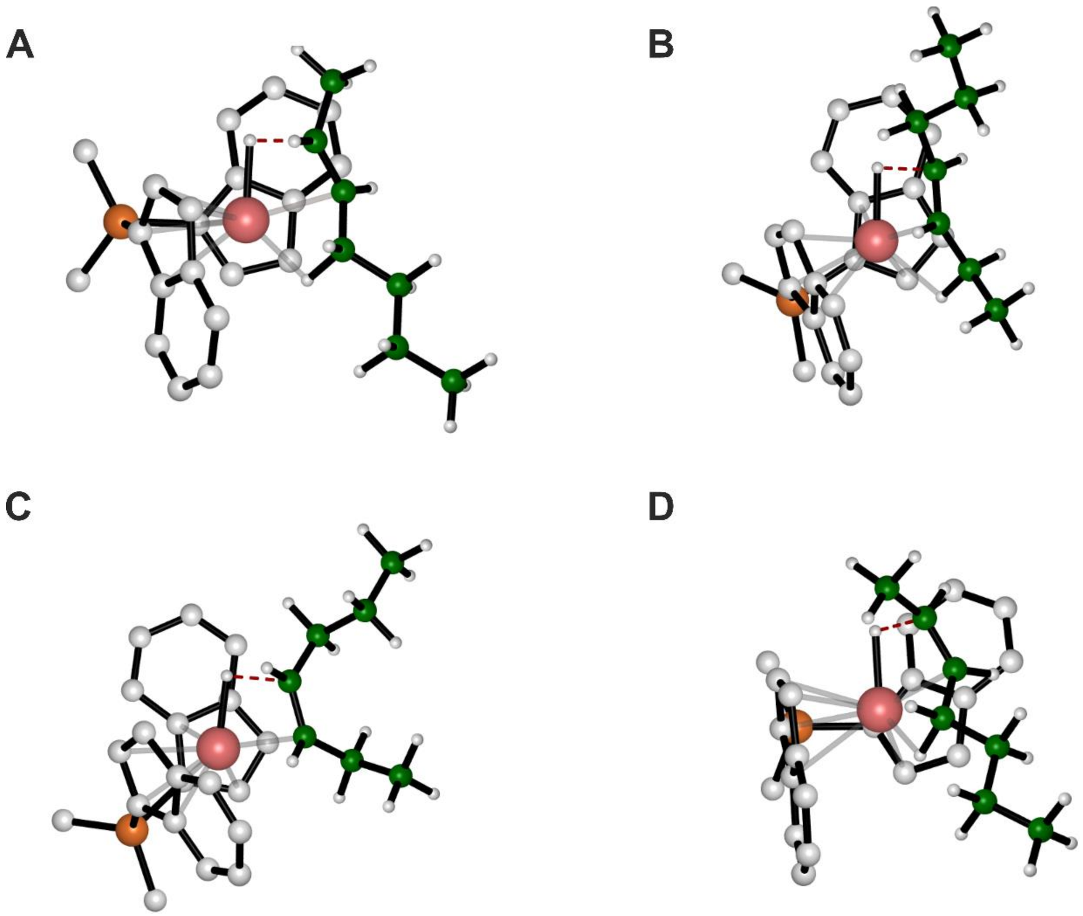

Figure S6. Optimized structures for the BHE TSs occurring after a 2,1 butene insertion. β-H transfer occurring from the methylene of the ethyl side group to the metal centre, the forming C=C is in *trans* and *cis* configurations in structures (A) and (D), respectively. β-H transfer occurring from the methylene of the main chain to the zirconium atom, the forming C=C is in *cis* and *trans* configurations in structures (C) and (B), respectively.

Table S1. Calculated $\Delta E(\Delta G)^{\#}$ in kcal/mol for the BHT TSs occurring after a 2,1 butene insertion, represented in Figure S3. The values are calculated with respect to the most stable BHT TS.

**BHT TSs**	$\mathbf{\Delta E(\Delta G)^{\#a)}}$
**A**	0.0 (0.0) kcal/mol
**B**	7.1 (6.7) kcal/mol
**C**	0.6 (1.1) kcal/mol
**D**	7.8 (7.1) kcal/mol

Table S2. Calculated $\Delta E(\Delta G)^{\#}$ in kcal/mol for the BHE TSs occurring after a 2,1 butene insertion, represented in Figure S4. The values are calculated with respect to the most stable BHE TS.

**BHE TSs**	$\mathbf{\Delta E(\Delta G)^{\#a)}}$
**A**	0.0 (0.0) kcal/mol
**B**	1.0 (0.8) kcal/mol
**C**	2.4 (2.1) kcal/mol
**D**	1.9 (1.9) kcal/mol

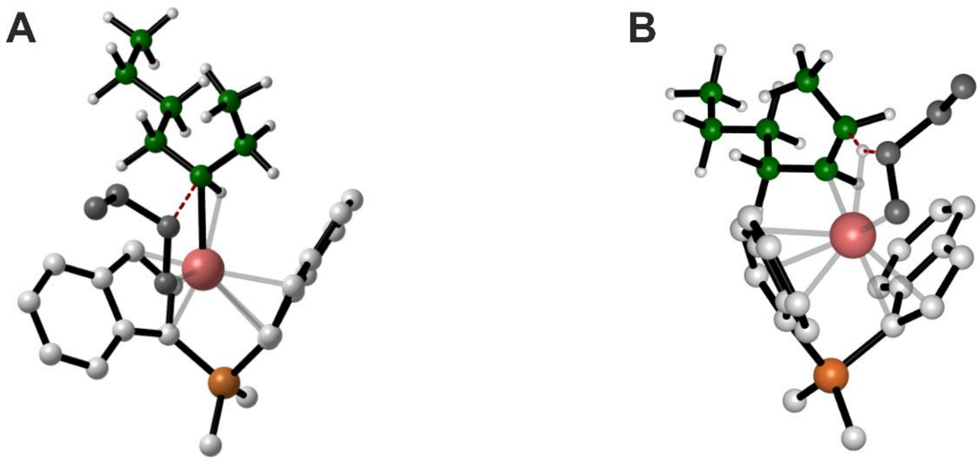

Figure S7. Optimized structures for the “right” (1,2 *si*) butene insertion into the epimerized secondary chain (A) and the favored BHT TS occurring after the epimerization of the secondary chain (B). β-H transfer occurs from the methylene of the ethyl side group to the 1,2 *re* butene enantioface with the forming C=C being in *cis* configuration.

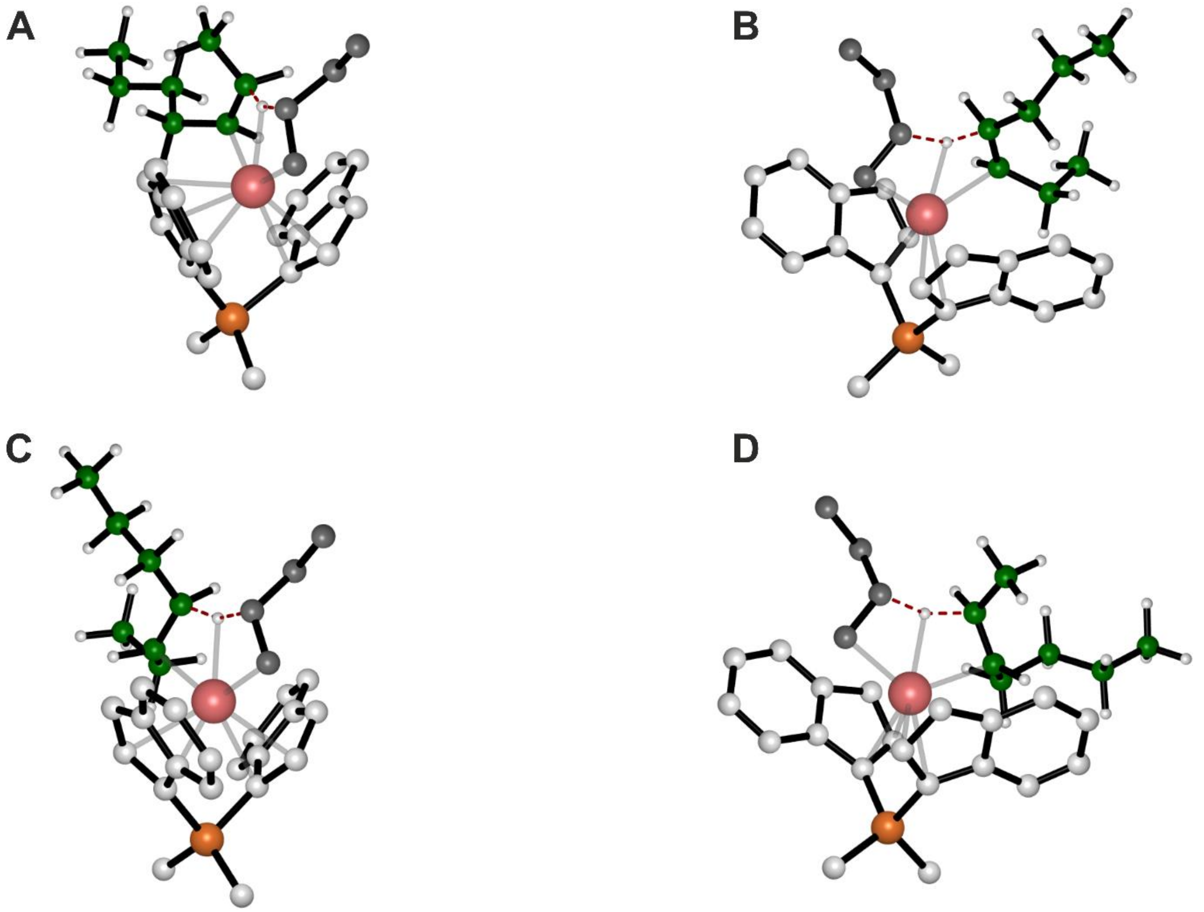

Figure S8. Optimized structures for all the BHT TSs occurring after the epimerization of the secondary chain. β-H transfers occurring from the methylene of the ethyl side group to the 1,2 re butene enantioface with the forming C=C being in *cis* and *trans* configurations correspond to structures (A) and (D), respectively. β-H transfer occurring from the methylene of the main chain to the zirconium atom with the forming C=C showing *cis* and *trans* configurations correspond to the structures (B) and (C), respectively.

Table S3. Calculated $\Delta E(\Delta G)^{\#}$ in kcal/mol for the BHT TSs occurring after the epimerization of the secondary chain, represented in Figure S5. The values are calculated with respect to the most stable BHT TS.

**BHT TSs**	**$\Delta E(\Delta G)^{\#a)}$**
**A**	0.0 (0.0) kcal/mol
**B**	8.3 (8.2) kcal/mol
**C**	7.4 (7.5) kcal/mol
**D**	3.4 (1.3) kcal/mol
